# A nomogram risk prediction model for no-reflow after primary percutaneous coronary intervention based on rapidly accessible patient data among patients with ST-segment elevation myocardial infarction and its relationship with prognosis

**DOI:** 10.3389/fcvm.2022.966299

**Published:** 2022-08-08

**Authors:** Yehong Liu, Ting Ye, Ke Chen, Gangyong Wu, Yang Xia, Xiao Wang, Gangjun Zong

**Affiliations:** ^1^Department of Cardiology, The 904th Hospital of Joint Logistic Support Force of PLA, Wuxi Clinical College of Anhui Medical University, Wuxi, China; ^2^The Fifth Clinical College of Anhui Medical University, Wuxi, China

**Keywords:** no-reflow, percutaneous coronary intervention, ST-segment elevation myocardial infarction, nomogram risk prediction model, prognosis, major adverse cardiovascular events

## Abstract

**Background:**

No-reflow occurring after primary percutaneous coronary intervention (PCI) in patients with ST-segment elevation myocardial infarction (STEMI) can increase the incidence of major adverse cardiovascular events (MACE). The present study aimed to construct a nomogram prediction model that can be quickly referred to before surgery to predict the risk for no-reflow after PCI in STEMI patients, and to further explore its prognostic utility in this patient population.

**Methods:**

Research subjects included 443 STEMI patients who underwent primary PCI between February 2018 and February 2021. Rapidly available clinical data obtained from emergency admissions were collected. Independent risk factors for no-reflow were analyzed using a multivariate logistic regression model. Subsequently, a nomogram for no-reflow was constructed and verified using bootstrap resampling. A receiver operating characteristic (ROC) curve was plotted to evaluate the discrimination ability of the nomogram model and a calibration curve was used to assess the concentricity between the model probability curve and ideal curve. Finally, the clinical utility of the model was evaluated using decision curve analysis.

**Results:**

The incidence of no-reflow was 18% among patients with STEMI. Killip class ≥2 on admission, pre-operative D-dimer and fibrinogen levels, and systemic immune–inflammation index (SII) were independent risk factors for no-reflow. A simple and quickly accessible prediction nomogram for no-reflow after PCI was developed. This nomogram demonstrated good discrimination, with an area under the ROC curve of 0.716. This nomogram was further validated using bootstrapping with 1,000 repetitions; the C-index of the bootstrap model was 0.706. Decision curve analysis revealed that this model demonstrated good fit and calibration and positive net benefits. Kaplan–Meier survival curve analysis revealed that patients with higher model scores were at a higher risk of MACE. Multivariate Cox regression analysis revealed that higher model score(s) was an independent predictor of MACE (hazard ratio 2.062; *P* = 0.004).

**Conclusions:**

A nomogram prediction model that can be quickly referred to before surgery to predict the risk for no-reflow after PCI in STEMI patients was constructed. This novel nomogram may be useful in identifying STEMI patients at higher risk for no-reflow and may predict prognosis in this patient population.

## Introduction

Cardiovascular disease is currently a key global public health concern and poses a significant threat to human health (1). Coronary artery disease is a type of atherosclerotic cardiovascular disease that has exhibited an unprecedented increase in incidence in some low- to middle-income countries, with a tendency of onset toward younger age (2). ST-segment elevation myocardial infarction (STEMI) is a serious type of coronary artery disease. Its pathogenesis involves an interrelated series of processes induced by early atherosclerosis and subsequent atherosclerotic plaque rupture. This condition is characterized by rapid progression, leading to complete occlusion of the culprit vessels, which can cause myocardial ischemic necrosis in the vascular territory, thus resulting in poor prognosis (3). The primary goals of treatment in patients with STEMI include recanalization of the occluded blood vessels and immediate restoration of coronary blood flow. Primary percutaneous coronary intervention (PCI) is the preferred method for early coronary reperfusion (4).

Studies have shown that ~10–30% of patients who undergo primary PCI experience the no-reflow phenomenon after recanalization of occluded blood vessel(s) (4). The no-reflow phenomenon refers to low or absent perfusion of myocardial tissues in the territory of the occluded coronary arteries after recanalization in patients with STEMI. The occurrence of this phenomenon can increase the extent of myocardial ischemic necrosis and the incidence of adverse cardiovascular events (e.g., in-hospital mortality, malignant arrhythmia, cardiogenic shock, and heart failure), as well as negate the cardiovascular benefits provided by the restoration of coronary blood flow in patients with STEMI (5). Existing studies suggest that the coagulation cascade resulting from microvascular dysfunction caused by capillary damage, endothelial cell swelling, changes in blood viscosity, oxidative damage, myocardial edema, thromboembolism, and distal microthrombosis after recanalization of occluded coronary arteries may be involved in the occurrence of coronary no-reflow after primary PCI (6). Furthermore, clinical studies have demonstrated that diabetes, age, sex, time from onset of chest pain to recanalization, pre-PCI thrombus score, collateral circulation, Killip class on admission, and elevated levels of inflammatory markers (e.g., neutrophil count) are associated with the occurrence of no-reflow after primary PCI (7, 8).

Current measures for the prevention and treatment of no-reflow after primary PCI are inadequate. As such, the construction of a rapid/easy-to-use and effective prediction model by analyzing risk factors for this phenomenon is a topic of interest in clinical research. For example, Wang et al. established a risk prediction model for no-reflow after primary PCI in patients with STEMI, which was based on blood biochemical parameters (e.g., liver and kidney function indicators), general clinical features of patients, and pre-operative medications (9). In addition, the same authors used blood indicators and intraoperative parameters of emergency coronary angiography to build a risk prediction model for no-reflow after primary PCI (8). Yang et al. established a risk prediction model for no-reflow after primary PCI in patients with STEMI, which was based on electrocardiogram changes at admission, general clinical features of patients, and intraoperative parameters of emergency coronary angiography (10). The establishment of these risk prediction models has played a positive role in the prevention of no-reflow after primary PCI. However, the measurement of some of the parameters included in these models, such as liver and kidney function indicators, requires at least 90 min. In clinical practice, a shorter door-to-balloon time—defined as the time from the emergency department arrival of a patient with chest pain to the diagnosis of STEMI and implementation of primary PCI—is beneficial. Accordingly, all chest pain centers in China have set a door-to-balloon time of <90 min. Therefore, the long data acquisition time for the parameters used in these prediction models restricts their implementation and practicality in clinical settings.

As such, the primary aim of the present study was to establish a rapid-to-use and stable nomogram prediction model for no-reflow after primary PCI in patients with STEMI based on parameters with a short acquisition time (<20 min), including routine blood test and coagulation function parameters, and general clinical features. In addition, this study aimed to evaluate the predictive value of this model for in-hospital and 1-year post-discharge prognosis of patients with STEMI who underwent primary PCI. We anticipate that this model will provide clinicians with a rapid-use, stable, and reliable tool for the early prediction and prevention of no-reflow after primary PCI in high-risk patients with STEMI, thereby improving the prognosis of this patient population.

## Methods

### Patients and grouping

Data from 473 patients with STEMI, who visited the Department of Cardiovascular Medicine of the 904th Hospital of the Chinese People's Liberation Army Joint Logistic Support Force and underwent primary PCI between February 2018 and February 2021, were retrospectively analyzed. After excluding specific patients, 443 (366 men and 77 women), with a mean age of 61 years (range, 50.5–69 years), were ultimately included in the study. The following individuals were excluded: STEMI patients who underwent thrombolytic therapy within 12 h of onset; patients with severe renal insufficiency, chronic or acute infection, inflammatory disease, coagulopathy, thrombocytosis or thrombocytopenia, malignant tumor or hematological disease, and allergy to contrast agents or anticoagulants; those who refused PCI; and those with incomplete clinical data. According to the Thrombolysis in Myocardial Infarction (TIMI) classification of coronary blood flow after percutaneous transluminal coronary angioplasty or stent placement, the 443 patients were divided into 2 groups: reflow [TIMI = 3 (*n* = 363)]; and no-reflow [TIMI <3 (*n* = 80)]. The incidence of no-reflow after primary PCI was 18%. This study was approved by the local ethics committee, and a written informed consent was obtained from the patients and their family members before the primary PCI procedure.

### Collection of clinical data

General clinical information and rapidly accessible pre-operative laboratory data of all patients were collected. General clinical information included the following: sex; age; height; weight; medical history; Killip class on admission; time from onset of chest pain to the PCI procedure; and systolic blood pressure, diastolic blood pressure, and heart rate on admission. Body mass index (BMI) was calculated using the patients' height and weight measurements. Laboratory data included routine blood test parameters, such as red blood cell count, hemoglobin level, white blood cell count, neutrophil count, monocyte count, lymphocyte count, platelet count, neutrophil percentage, lymphocyte percentage, platelet distribution width, mean platelet volume, and mean corpuscular volume, as well as coagulation function parameters including D-dimer level, fibrinogen level, international normalized ratio (INR), and prothrombin activity (PTA). Calculations were also performed in accordance with the appropriate formula to obtain the systemic immune–inflammation index (SII; neutrophil count × platelet count/lymphocyte count), mean platelet volume-to-lymphocyte ratio (mean platelet volume/lymphocyte count), ratio of platelet to mean corpuscular volume (platelet count/mean corpuscular volume), and monocyte-to-lymphocyte ratio (MLR; monocyte count/lymphocyte count).

### Percutaneous coronary angiography and definition of post-operative no-reflow

All patients with STEMI underwent pre-operative treatment according to clinical guideline recommendations, which included administration of chewable 300 mg enteric-coated aspirin tablets and 180 mg ticagrelor. Two specialists performed coronary angiography in accordance with the standard Judkins technique (11). These specialists decided on the specific PCI procedure based on the actual condition of the culprit vessels. After recanalization, the two specialists evaluated the status of coronary blood flow (TIMI flow grade) based on findings from coronary angiography. TIMI grades 0, 1, and 2 were defined as no-reflow, whereas TIMI grade 3 was defined as reflow (12).

### Construction and validation of the nomogram risk prediction model

After comparing clinical data between the no-reflow and reflow groups, independent risk factors for no-reflow after primary PCI among STEMI patients were identified using univariate and multivariate logistic regression analysis. Subsequently, a nomogram model was constructed based on the results of multivariate logistic regression using the generalized linear model (i.e., “glm”) function in R version 4.1.2 (R Foundation for Statistical Computing, Vienna, Austria).

After calculating the total score for each patient based on the nomogram risk prediction model, receiver operating characteristic (ROC) curve analysis was performed to determine the discrimination ability of the nomogram model. The Hosmer–Lemeshow goodness of fit test was used to determine the agreement between the probability that the nomogram model predicted no reflow after primary PCI and the actual probability.

Internal bootstrap validation was used with repeated sampling (1,000 repetitions) to verify the accuracy of the nomogram model. Decision curve analysis (DCA) was used to evaluate the clinical validity of nomogram model.

### In-hospital and 1-year post-discharge major adverse cardiovascular events after primary PCI in patients with STEMI

In-hospital major adverse cardiovascular events (MACE) included new-onset cardiac death, non-fatal cerebral and myocardial infarction, cardiogenic shock, heart failure, malignant arrhythmia (ventricular fibrillation and tachycardia), high-grade atrioventricular block, new-onset atrial fibrillation, and gastrointestinal hemorrhage after primary PCI. The primary endpoints at 1-year post-discharge included cardiac death, new-onset non-fatal myocardial infarction, rehospitalization for malignant arrhythmia, unstable angina (stent implantation), and heart failure. Follow-up information was collected on rehospitalization and through telephone contact or outpatient visits at 6 months and 1 year.

### Statistical analysis

The Kolmogorov–Smirnov test was used to determine whether continuous variables conformed to a normal distribution and, for those that did, are expressed as mean ± standard deviation. Those that did not conform to a normal distribution are expressed as median (interquartile range. i.e., 25th−75th percentile [IQR]). Classification variables are expressed as number or percentage. The Student's *t*-test, Mann–Whitney *U* test, and chi-squared test were used to identify significant differences among the groups. Spearman correlation coefficients were used for correlation analysis. Multicollinearity of variables was tested using the variance inflation factor (VIF), with VIF >10 considered to be indicative of multicollinearity. Multivariable logistic regression analyses were performed to identify independent predictors of no-reflow. The De-Long test was used to compare the ROC curves for Killip class on admission, fibrinogen and D-dimer levels, SII/100 level, and nomogram model. ROC curve analysis was used to determine the optimal cut-off value of the nomogram model to predict MACE. Kaplan–Meier curves and Cox proportional hazard regression models were used to analyze the relationship between the clinical data and 1-year post-discharge MACE. All tests were two-tailed and differences with *P* < 0.05 were considered to be statistically significant.

## Results

### Basic characteristics

With regard to general clinical information (Table 1), there were no statistically significant differences (*P* > 0.05) between the reflow and no-reflow groups in terms of sex, age, BMI, medical history (hypertension, diabetes, smoking, statin use, antiplatelet therapy, angiotensin-converting enzyme inhibitor/angiotensin receptor blocker use, calcium channel blocker use, and β-blocker use), systolic/diastolic blood pressure on admission, heart rate, routine blood test parameters (white blood cell count, red blood cell count, and hemoglobin level), and coagulation function parameters (PTA and INR). The no-reflow group exhibited poorer cardiac function on admission (Killip class ≥ 2) and a longer time from onset of chest pain to PCI compared with the reflow group. The no-reflow group also differed significantly from the reflow group in terms of SII/100, and fibrinogen and D-dimer levels (*P* < 0.001). In addition, neutrophil count (*P* = 0.017), neutrophil percentage (*P* = 0.025), monocyte count (*P* = 0.040), platelet count (*P* = 0.046), and MLR (*P* < 0.001) were significantly higher, whereas lymphocyte count (*P* = 0.040) and lymphocyte ratio (*P* = 0.003) were lower in the no-reflow group than in the reflow group.

**Table 1:** Basic clinical characteristics of no-reflow group and reflow group.

**Variable**	**No-reflow (*n* = 80)**	**Reflow (*n* = 363)**	***P-*value^b^**
Men, *n* (%)	65 (81)	301 (83)	0.721
Hypertension, *n* (%)	53 (66)	202 (56)	0.082
Diabetes mellitus, *n* (%)	16 (20)	82 (23)	0.613
Smoking, *n* (%)	50 (63)	240 (66)	0.538
**Pre-procedural medications**
Statins, *n* (%)	3 (4)	10 (3)	0.633
Aspirin or Clopidogrel, *n* (%)	4 (5)	16 (4)	0.817
ACEI or ARB, *n* (%)	17 (21)	63 (17)	0.412
CCB, *n* (%)	20 (25)	82 (23)	0.634
β-blocker, *n* (%)	5 (6)	17 (5)	0.559
Admission killip class ≥2, *n* (%)	29 (36)	63 (17)	<0.001
Pain to PCI time, *h*	3.5 (2.5, 6)	3 (2, 5)	0.040
Age, years	64 (49, 70)	60 (51, 69)	0.342
BMI (kg/m^2^)	24.17 (22.65, 25.35)	24.22 (22.53, 25.83)	0.517
Systolic blood pressure (mmHg)	130 (118, 146)	129 (120, 146)	0.980
Diastolic blood pressure (mmHg)	80 (70, 91)	80 (70, 88)	0.872
Heart rate (bpm)	70 (64, 85)	72 (68, 86)	0.530
Mean corpuscular volume (fl)	91 (87, 94)	90 (87, 93)	0.462
Hemoglobin (g/dL)	137 (123, 147)	139 (127, 149)	0.374
Red blood cell (10^3^/ul)	4.411 ± 0.655	4.476 ± 0.560	0.361^a^
Packed cell volume (l/l)	0.399 ± 0.053	0.402 ± 0.048	0.633^a^
Mean corpuscular hemoglobin (pg)	30.80 (29.93, 32.25)	30.70 (29.60, 31.90)	0.283
Neutrophil ratio (%)	0.782 (0.722, 0.833)	0.760 (0.684, 0.824)	0.025
Lymphocyte ratio (%)	0.140 (0.098, 0.177)	0.160 (0.108, 0.218)	0.003
Basophil ratio (%)	0.002 (0.001, 0.003)	0.002 (0.001, 0.004)	0.602
Neutrophil (10^3^/ul)	8.255 (6.268, 10.268)	7.290 (5.160, 9.620)	0.017
Monocytes ratio (%)	0.074 (0.055, 0.097)	0.071 (0.056, 0.090)	0.429
Lymphocyte (10^3^/ul)	1.315 (0.983, 1.880)	1.540 (1.120, 2.030)	0.040
Eosinophil (10^3^/ul)	0.030 (0.010, 0.060)	0.040 (0.010, 0.100)	0.380
Monocytes (10^3^/ul)	0.750 (0.523, 0.988)	0.640 (0.480, 0.870)	0.040
Mean platelet volume (fl)	10.8 (10.2, 11.5)	10.9 (10.3, 11.6)	0.328
Platelet distribution width (%)	12.4 (11.1, 14.1)	12.9 (11.7, 14.8)	0.067
Platelet (10^3^/ul)	216 ± 60	202 ± 56	0.046^a^
Eosinophil ratio (%)	0.003 (0.001, 0.007)	0.004 (0.001, 0.012)	0.326
White blood cell (10^3^/ul)	10.78 (7.97, 13.51)	9.97 (7.54, 12.24)	0.240
Basophil (10^3^/ul)	0.02 (0.01, 0.03)	0.02 (0.01, 0.03)	0.990
Plateletcrit (%)	0.24(0.18, 0.27)	0.22(0.19, 0.26)	0.180
Mean corpuscular hemoglobin concentration (g/L)	342.388 ± 12.494	340.669 ± 11.221	0.225^a^
D-dimer (mg/L)	0.53 (0.31, 0.90)	0.31 (0.20, 0.56)	<0.001
Prothrombin time activity (%)	102.4 (88.0, 116.2)	102.4 (89.4, 116.3)	0.776
International normalized ratio (%)	1.06 (0.99, 1.15)	1.06 (0.99, 1.14)	0.693
Fibrinogen (g/L)	3.50 (2.80, 4.11)	2.96 (2.43, 3.45)	<0.001
SII/100 (10^3^/ul)	11.78 (8.70, 18.13)	8.71 (5.61, 15.18)	<0.001
MPVLR	8.46 (5.71, 10.46)	7.31 (5.40, 9.92)	0.091
P/MCV	2.41 (1.88, 2.74)	2.24 (1.83, 2.67)	0.088
MLR	0.56 (0.38, 0.77)	0.43 (0.32, 0.57)	<0.001

Values are presented as mean ± SD, number (%) or median (interquartile range). Systemic immune-inflammation index (SII, neutrophil count * platelet count/lymphocyte count); Mean platelet volume to lymphocyte ratio (MPVLR, mean platelet volume/lymphocyte count); Platelet/mean corpuscular volume (P/MCV), and Monocyte-to-lymphocyte ratio (MLR, monocyte count/lymphocyte count).

a *Unpaired Student's t test.*

b *Mann–Whitney U test and chi-square test*.

**Figure 1:**
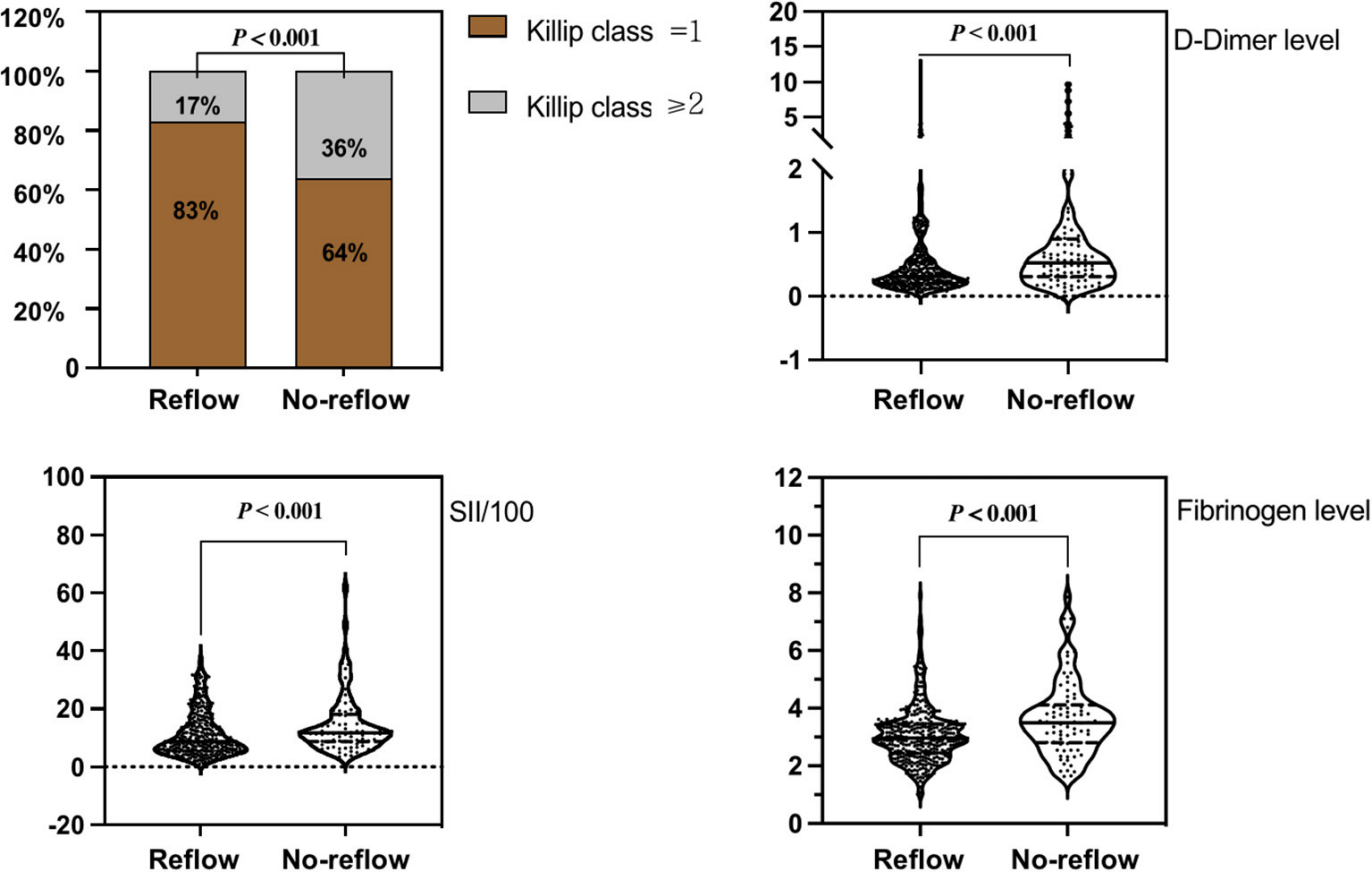
Comparison of Killip class on admission, D-dimer level, fibrinogen level, and SII/100 values between groups.

Correlation analysis revealed that SII/100 was correlated with neutrophil count (*r* = 0.773, *P* < 0.05), neutrophil percentage (*r* = 0.863, *P* < 0.05), and lymphocyte percentage (*r* = −0.860, *P* < 0.05), whereas neutrophil ratio was correlated with lymphocyte ratio (*r* = −0.942, *P* < 0.05). No multicollinearity was found for the other indicators (VIF <10). Therefore, SII/100, MLR, platelet count, lymphocyte count, fibrinogen and D-dimer levels, Killip class on admission, and time from onset of chest pain to PCI were included in the multivariate logistic regression analysis. As shown in Table 2, Killip class on admission odds ratio {OR 1.839 [95% confidence interval (CI)] 1.025–3.298; *P* = 0.041}, D-dimer level [OR 1.218 (95% CI 1.007–1.472); *P* = 0.042], fibrinogen level [OR 1.473 (95% CI 1.186–1.829); *P* < 0.001], and SII/100 [OR 1.034 (95% CI 1.005–1.063); *P* = 0.019] were independent risk factors for no-reflow after primary PCI among patients with STEMI. Given the differences in Killip class on admission, D-dimer and fibrinogen levels, and SII/100 between the two groups (Figure 1), Spearman correlation analysis revealed that SII/100 was significantly and positively correlated with Killip class on admission (*r* = 0.097, *P* = 0.041) and D-dimer level (*r* = 0.188, *P* < 0.001) and demonstrated no linear correlation with fibrinogen level (*r* = 0.06, *P* = 0.901). D-dimer level was positively correlated with fibrinogen level (*r* = 0.158, *P* = 0.001) and Killip class on admission (*r* = 0.306, *P* < 0.001), and fibrinogen level was positively correlated with Killip class on admission (*r* = 0.130, *P* = 0.06).

**Table 2:** Independent predictors of no-reflow was determined by multivariate logistic regression analysis.

**Independent predictors of no-reflow**							
	**Univariate analysis**				**Multivariable analysis**			
	***P-*** **value**	**OR**	**95% CI**		***P-*** **value**	**OR**	**95% CI**	
Platelet (10^3^/ul)	0.047	1.004	1.000	1.009	–	–	–	–
Pain to PCI time, *h*	0.016	1.118	1.021	1.224	–	–	–	–
Lymphocyte (10^3^/ul)	0.019	0.627	0.425	0.926	–	–	–	–
Monocytes (10^3^/ul)	0.075	1.803	0.942	3.450	–	–	–	–
MLR	0.003	3.391	1.502	7.657	–	–	–	–
Admission killip class ≥2, *n* (%)	<0.001	2.708	1.593	4.603	0.041	1.839	1.025	3.298
D-dimer (mg/L)	0.004	1.311	1.088	1.580	0.042	1.218	1.007	1.472
Fibrinogen (g/L)	<0.001	1.557	1.264	1.917	<0.001	1.473	1.186	1.829
SII/100 (10^3^/ul)	0.001	1.043	1.017	1.071	0.019	1.034	1.005	1.063

Systemic immune-inflammation index (SII, neutrophil count * platelet count/lymphocyte count); Monocyte-to-lymphocyte ratio (MLR, monocyte count/lymphocyte count).

### Construction of the nomogram risk prediction model

**Figure 2:**
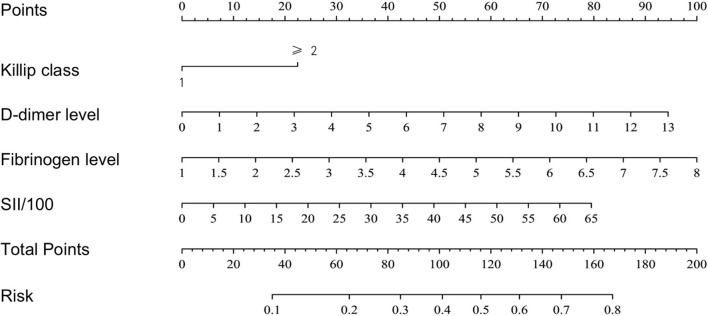
The nomogram model for predicting the risk of no-reflow after PCI in STEMI patients.

Based on the results of the multivariate logistic regression analysis, a nomogram risk prediction model with four significant risk factors to predict the risk for no-reflow after primary PCI among patients with STEMI (Figure 2) was constructed. Each index corresponds to a score in the top point line, and then the total point score is the sum of the four index scores. The total point score is projected on the bottom scales to judge the probability of no-reflow after PCI in STEMI patients.

### Validation of the nomogram risk prediction model

**Figure 3:**
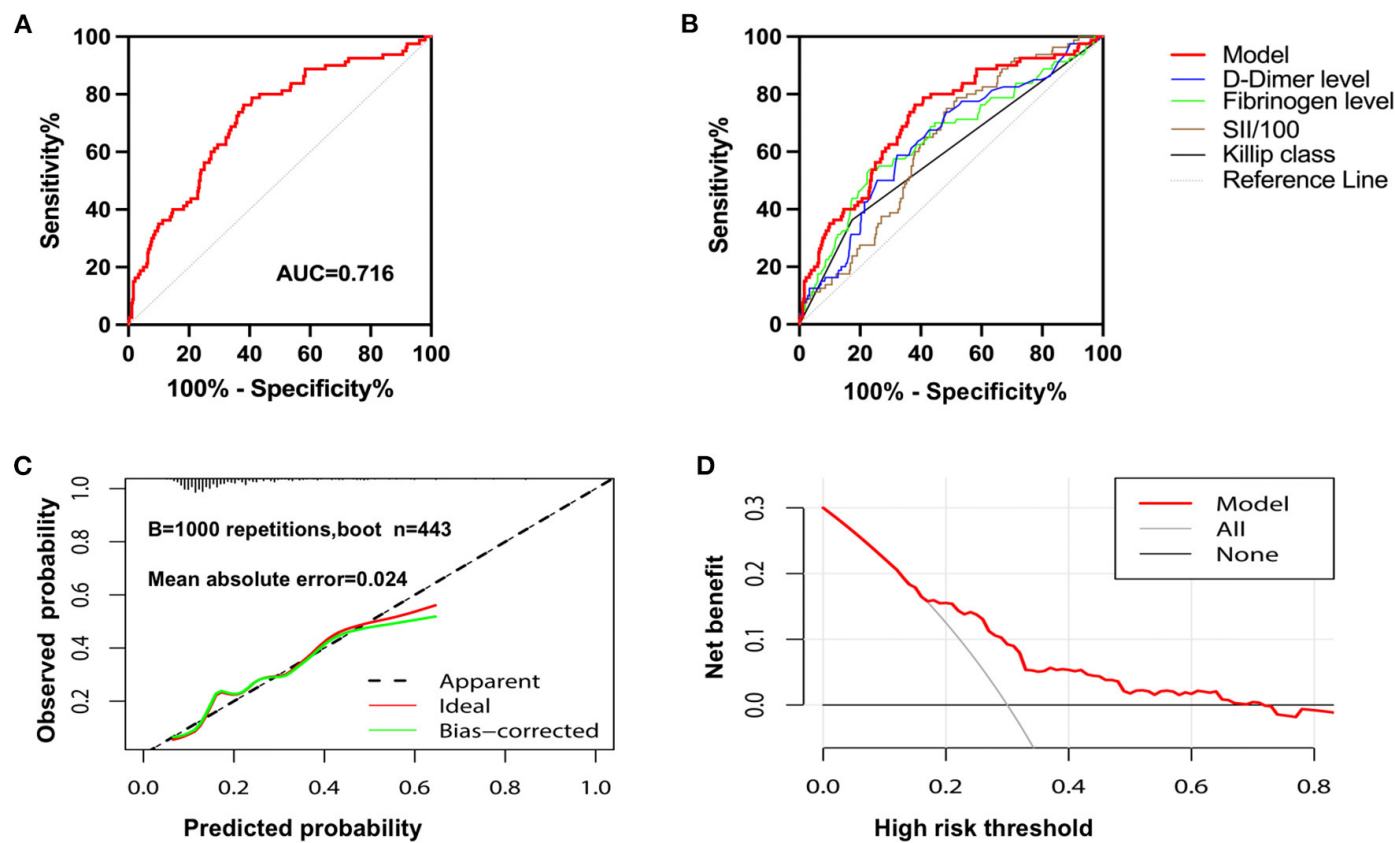
The evaluation of the nomogram model. **(A)** Receiver operating characteristic curve for assessing the discrimination performance of the nomogram model. **(B)** Receiver operating characteristic curves of patients with no-reflow predicted by Killip class on admission, D-dimer, fibrinogen, SII/100 and nomogram model. **(C)** Calibration curve of nomogram model in predicting the risk of no-reflow. **(D)** The decision curve analysis of the nomogram model.

After calculating the total score for each patient based on the nomogram risk prediction model, ROC curve analysis was performed to determine the discrimination ability of the nomogram model, with an area under the ROC curve (AUC) of 0.716 (95% CI 0.654–0.779) (Figure 3A). The diagnostic performance of the nomogram risk prediction model was superior to that of D-dimer level [AUC 0.716 (95% CI 0.654–0.779) vs. AUC 0.639 (95% CI 0.507–0.707); *P* = 0.044], fibrinogen level [AUC 0.716 (95% CI 0.654–0.779) vs. AUC 0.648 (95% CI 0.577–0.719); *P* = 0.015], SII/100 [AUC 0.716 (95% CI 0.654–0.779) vs. AUC 0.630 (95% CI 0.569–0.691); *P* = 0.020], and Killip class on admission [AUC 0.716 (95% CI 0.654–0.779) vs. AUC 0.594 (95% CI 0.538–0.651); *P* < 0.001] (Figure 3B).

The Hosmer–Lemeshow goodness of fit test revealed that the deviation between the risk prediction value of the nomogram model and the actual observed value demonstrated no statistical significance (χ^2^ = 11.325, df = 8, *P* = 0.1839). This implies that the nomogram model had a good fit, and its prediction of the probability of no-reflow after primary PCI demonstrated good concordance with the actual probability.

Internal bootstrap validation was used with repeated sampling (1,000 repetitions) to verify the nomogram model. The C-index of the bootstrap nomogram model was 0.706, with a discrimination ability similar to that of the initial nomogram model. The internal bootstrap validation calibration curve demonstrated that the mean absolute error of the calibration curve was 0.024, indicating that the calibration curve was in good agreement with the ideal curve (Figure 3C).

DCA of the nomogram model is shown in Figure 3D. When the predicted risk of no-reflow after PCI was 0.15–0.72, more significant net benefits were gained when treatment measures were implemented after primary PCI in patients with STEMI identified using this model than when no treatment was applied. The nomogram model for predicting no-reflow demonstrated the greatest benefit when the predicted risk for no-reflow after PCI was between 0.15 and 0.72.

### In-hospital MACE

**Figure 4:**
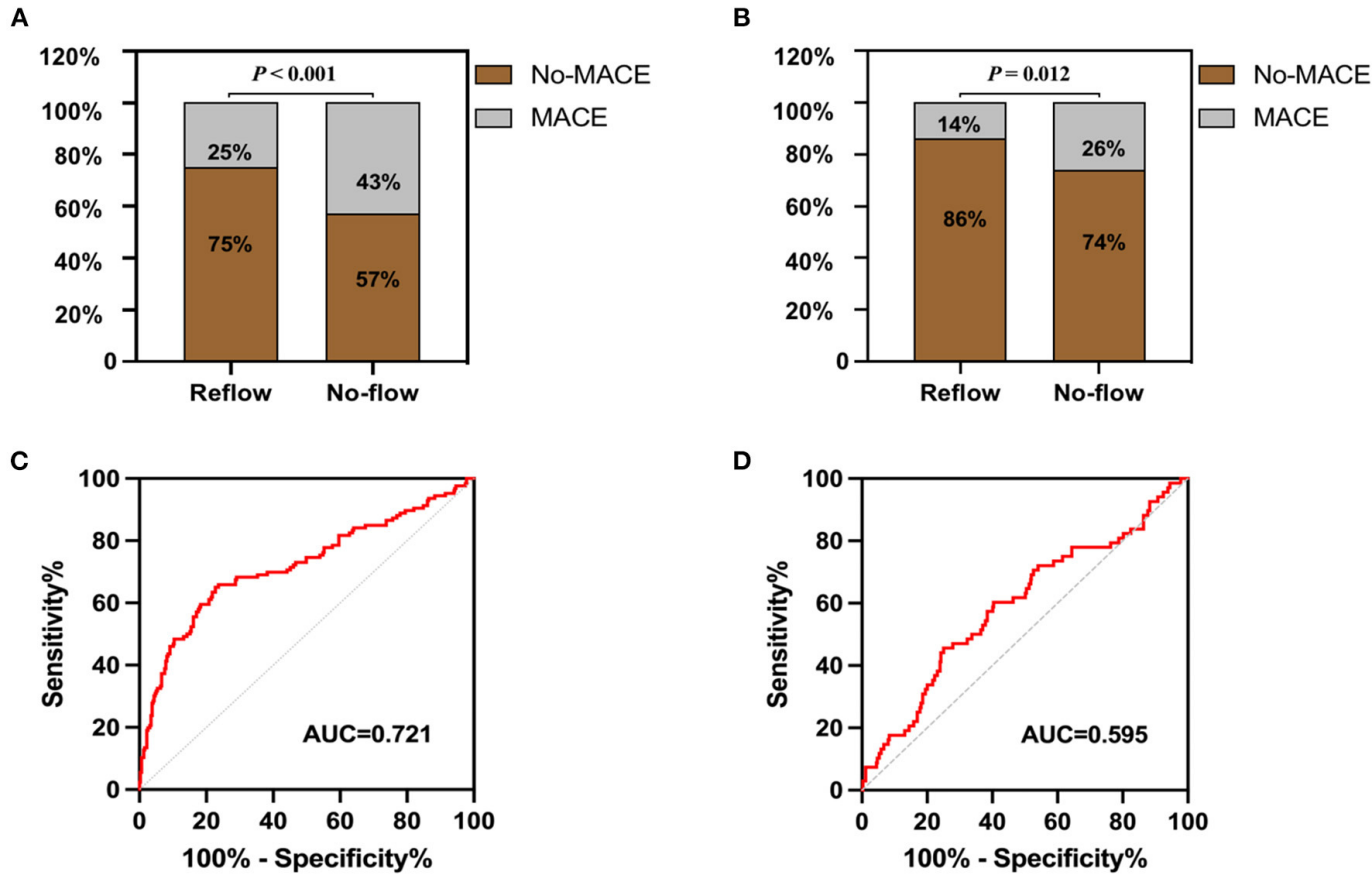
In-hospital and 1-year post-discharge MACE after primary PCI in patients with STEMI. **(A)** Comparison of MACE in hospital between groups. **(B)** Comparison of MACE in 1 year after discharge between groups. **(C)** Receiver operating characteristic curve of nomogram model for predicting the occurrence of in-hospital MACE. **(D)** Receiver operating characteristic curve of nomogram model for predicting the occurrence of 1-year post-discharge MACE.

The incidence of in-hospital MACE among patients with STEMI was 28.4%, and the cardiac death rate was 3.6%. The incidence of in-hospital MACE among patients who underwent primary PCI was higher in the no-reflow group than in the reflow group [*n* = 34 (43%) vs. *n* = 92 (25%); *P* < 0.001] (Figure 4A). More specifically, the mortality rate in the no-reflow group was higher than that in the reflow group (*P* = 0.039), and the incidence of non-fatal cardiovascular events was also higher in the no-reflow group than in the reflow group (*P* = 0.020) (Table 3). A ROC curve was used to determine the optimal cut-off value of the nomogram model to predict MACE. When the total score of the nomogram risk prediction model was >58, its diagnostic performance in predicting the occurrence of in-hospital MACE was 0.721 (95% CI 0.664–0.779; *P* < 0.001) (Figure 4C). The sensitivity of the nomogram model was 65.08%, and the specificity was 77.29%.

**Table 3:** In-hospital major adverse cardiac events by study group.

		**No-reflow (80)**	**Reflow (363)**	***P-*value**
In-hospital MACE:				<0.001
New-onset cardiac death		6 (7.50%)	10 (2.75%)	0.039
Non-fatal cardiovascular events		28 (35.00%)	82 (22.59%)	0.020
	Cerebral and myocardial infarction	0	2	
	Cardiogenic shock	4	5	
	Malignant arrhythmia	4	11	
	Heart failure	8	37	
	High-grade atrioventricular block	7	9	
	New-onset atrial fibrillation	5	16	
	Gastrointestinal hemorrhage	0	2	

*MACE, major adverse cardiac events; Malignant arrhythmia, ventricular fibrillation and tachycardia*.

### One-year post-discharge MACE

**Figure 5:**
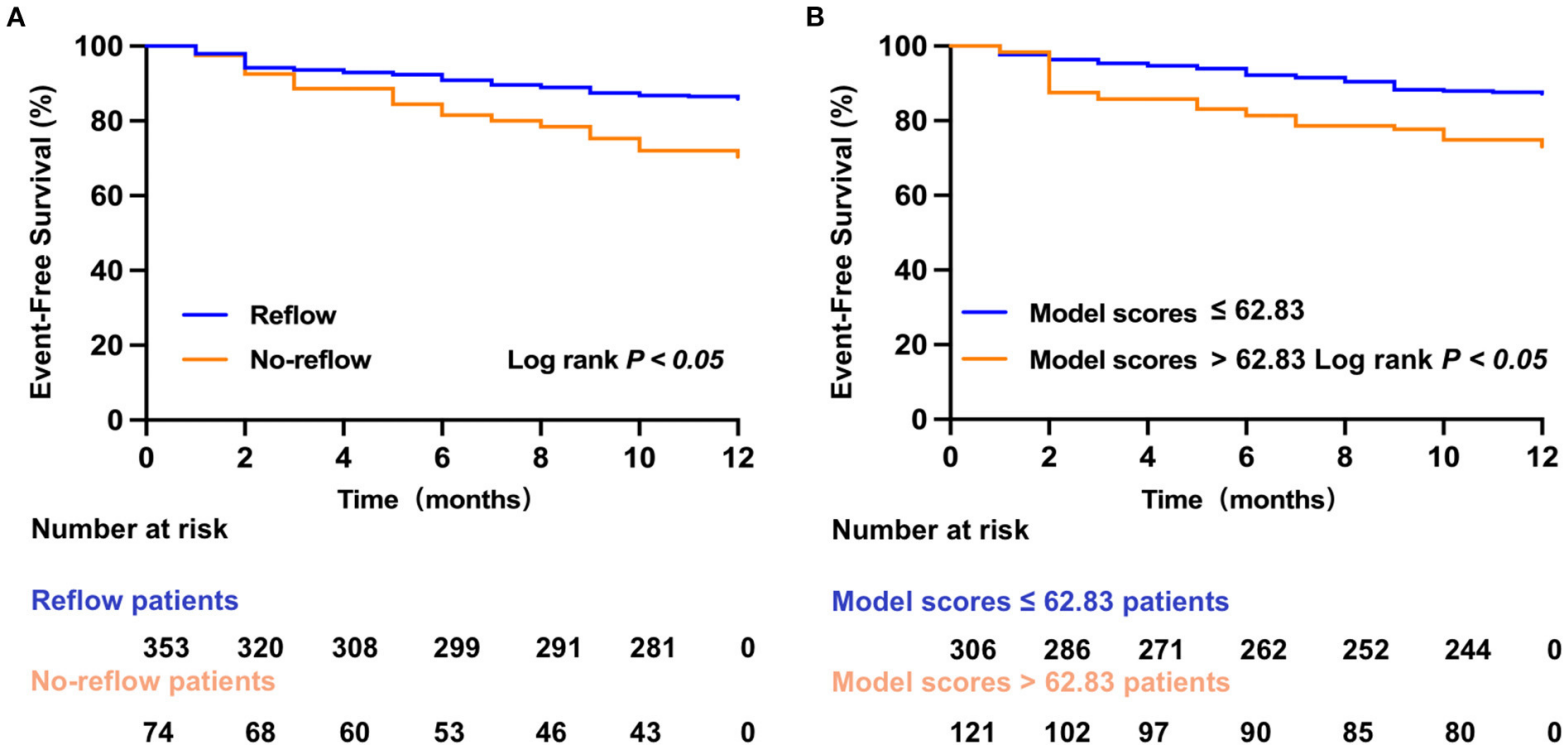
Kaplan–Meier survival analysis. **(A)** The event-free survival for MACE in reflow and no-reflow group. **(B)** The event-free survival for MACE in low and high nomogram model scores group.

A total of 68 STEMI patients developed MACE within 1 year after discharge, including: cardiac death (*n* = 5); new-onset non-fatal myocardial infarction (*n* =13); malignant arrhythmias (*n* = 2); heart failure (*n* = 21); and unstable angina (stent reimplantation) (*n* = 27). The incidence of MACE at 1-year post-discharge after primary PCI among patients with STEMI was higher in the no-reflow group than in the reflow group [*n* = 19 (26%) vs. *n* = 49 (14%); *P* = 0.012] (Figure 4B). Kaplan–Meier analysis was performed to compare the event-free survival rate of patients in the reflow and no-reflow groups. The event-free survival rate after primary PCI among patients with STEMI was significantly lower in the no-reflow group than in the reflow group (Figure 5A). A ROC curve was used to determine the optimal cut-off value of the nomogram model to predict MACE. When the cut-off value for the total score of the nomogram risk prediction model was 62.83, its predictive performance for the occurrence of MACE at 1-year post-discharge among patients with STEMI was 0.595, with a sensitivity of 45.59% and a specificity of 74.93% (Figure 4D). STEMI patients with a total score >62.83 in the nomogram risk prediction model exhibited a significantly lower event-free survival rate at 1-year post-discharge than those with a total score ≤ 62.83 (Figure 5B). A Cox proportional hazard regression model was used to identify independent risk factors for the occurrence of MACE at 1-year post-discharge among patients with STEMI. After adjusting for confounding factors (e.g., occurrence of post-PCI no-reflow and in-hospital MACE), a total score >62.83 in the nomogram risk prediction model remained a strong independent predictor of MACE at 1-year post-discharge in patients with STEMI (Table 4).

**Table 4:** The Cox proportional hazard regression model was used to determine the independent risk factors for the occurrence of MACE at 1-year post-discharge among patients with STEMI.

**Cox survival analysis of the predictors of MACE**								
	**Univariate analysis**				**Multivariable analysis**			
	***P-*** **value**	**HR**	**95% CI**		***P-*** **value**	**HR**	**95% CI**	
Lymphocyte ratio (%)	0.024	0.566	0.346	0.927	–	–	–	–
MLR	0.028	1.732	1.062	2.824	–	–	–	–
In-hospital MACE, *n* (%)	0.014	1.851	1.135	3.019	–	–	–	–
No-reflow, *n* (%)	0.008	2.047	1.204	3.479	0.071	1.662	0.958	2.881
D-dimer level (mg/L)	0.026	1.742	1.068	2.840	–	–	–	–
Admission Killip class ≥2, *n* (%)	0.041	1.739	1.023	2.954	–	–	–	–
High model scores (>62.83)	0.001	2.297	1.425	3.702	0.004	2.062	1.257	3.384

*MLR, monocyte count/lymphocyte count; MACE, major adverse cardiac events*.

## Discussion

The no-reflow phenomenon is a serious complication of primary PCI among patients with STEMI, and its occurrence significantly increases the incidence of MACE, including in-hospital and long-term mortality. Identifying patients at high risk for no-reflow before primary PCI would enable the necessary/appropriate intraoperative intervention(s) (e.g., thrombus aspiration or reduction of the number of pre-dilations) and the advanced provision of medication assistance (e.g., intracoronary administration of vasodilators such as adenosine, verapamil, and sodium nitroprusside). These measures would significantly contribute to the prevention of post-PCI no-reflow, which in turn would improve prognosis (13).

The pathophysiological mechanisms underlying the occurrence of no-reflow after primary PCI among patients with STEMI remain poorly understood. However, a mounting body of evidence suggests that the systemic immune–inflammatory response and distal microthrombosis after the recanalization of occluded vessels are involved in the occurrence of post-PCI no-reflow, and that the coagulation system plays a major role in thrombosis (6, 14). Therefore, the primary focus of the present study was to determine whether a nomogram risk prediction model for the early identification of no-reflow after primary PCI among patients with STEMI could be constructed using routine blood test parameters, coagulation function parameters, and general patient information, which are more rapidly and readily accessible than blood biochemical parameters and coronary angiographic findings in clinical practice. In addition, this study aimed to investigate the relationship between the performance of the nomogram model and the prognosis of patients with STEMI.

In the present study, univariate and multivariate logistic regression analyses were first performed to screen for independent risk factors for no-reflow after primary PCI among patients with STEMI. These included Killip class ≥2 on admission, D-dimer and fibrinogen levels, and SII/100. Based on these risk factors, a nomogram risk prediction model for no-reflow after PCI was constructed, which demonstrated good discrimination, calibration, and clinical validity. We further investigated the relationship between no-reflow after primary PCI among patients with STEMI and the nomogram risk prediction model and the incidence of in-hospital and 1-year post-discharge MACE. Findings revealed that the occurrence of no-reflow increased the incidence of in-hospital and 1-year post-discharge MACE, which was consistent with results reported in previous studies (15). The nomogram model demonstrated good predictive performance for in-hospital and 1-year post-discharge MACE after primary PCI in patients with STEMI when the total score was >58 and 62.83, respectively. Furthermore, after adjusting for confounding factors, such as clinical comorbidities, general patient information, in-hospital MACE, and post-PCI no-reflow, a total score >62.83 in the nomogram risk prediction model remained an independent risk factor for MACE at 1-year post-discharge after primary PCI in patients with STEMI [hazard ratio 2.062 (95% CI 1.257–3.384); *P* = 0.004]. The incidence of 1-year post-discharge MACE among STEMI patients who scored >62.83 was 2.062 times that of those who scored ≤ 62.83.

Inflammation and platelet aggregation play dominant roles in the pathophysiology of no-reflow after primary PCI among patients with STEMI (16). Inflammatory cell counts, such as neutrophils, platelets, and lymphocytes, play a crucial role in coronary atherosclerosis and myocardial infarction, among these, the neutrophil-to-lymphocyte ratio (NLR) and platelet-to-lymphocyte ratio (PLR) have been shown to be strong predictors of coronary no-reflow after primary PCI among patients with STEMI (17). The SII is a new inflammatory marker that reflects changes in neutrophil, platelet, and lymphocyte counts. Previous studies have reported that SII is positively correlated with the severity of coronary artery disease and that a high SII is indicative of poor prognosis among patients with coronary artery disease after coronary stent placement (18). Esenboga et al. reported that SII was an independent risk factor for no-reflow after primary PCI among patients with STEMI, and its predictive performance was superior to that of traditional predictors of no-reflow, such as NLR and PLR (19).

Fibrinogen level is a biomarker of chronic inflammatory response and is mainly involved in pathophysiological processes such as coagulation, fibrinolysis, and fibrin and platelet aggregation. Previous studies have demonstrated that an elevated plasma fibrinogen level is a risk factor for coronary artery lesions (20). Among patients with acute coronary syndrome, plasma fibrinogen level was positively correlated with the severity of coronary artery lesions (SYNTAX scores), whereas an elevated plasma fibrinogen level was an independent risk factor for moderate to severe coronary artery lesions (SYNTAX scores ≥23) (20). Zhao et al. reported that plasma fibrinogen level was an independent predictor of no-reflow after primary PCI among patients with STEMI, and a fibrinogen-to-albumin ratio ≥10.89 indicated poor prognosis after primary PCI in these patients (21). On the one hand, fibrinogen may be involved in the occurrence of no-reflow after primary PCI in patients with STEMI by contributing to inflammation of the vascular wall, leading to vascular endothelial injury (22). On the other hand, elevated fibrinogen levels can promote coagulation and accelerate platelet aggregation, which can result in hypercoagulability and accelerate microthrombosis (23).

Fibrinogen is first converted into fibrin monomers that aggregate to form fibrin polymers, which are subsequently degraded by plasmin during fibrinolysis. As a degradation product of fibrin polymers, D-dimer can serve as a marker of hypercoagulability and thrombotic events (24). Erkol et al. demonstrated that plasma D-dimer level was associated with the occurrence of no-reflow after primary PCI among patients with STEMI (25). Moreover, Zhang et al. reported that plasma D-dimer level can serve as a diagnostic marker for the occurrence of no-reflow after primary PCI in patients with STEMI (26).

Our results demonstrated that Killip class ≥2 on admission was an independent risk factor for no-reflow after primary PCI in patients with STEMI. The Killip classification, proposed by Killip et al. in 1967, is a useful method for early risk stratification of patients who experience acute myocardial infarction. A higher Killip class on admission was associated with in-hospital and 1-year mortality, thus suggesting poor prognosis (27). Furthermore, Wang et al. found that Killip class was an independent risk factor for the occurrence of no-reflow after primary PCI among patients with STEMI (9).

### Limitations

The present investigation was a single-center retrospective study with a small sample size and a relatively short follow-up period. Although our nomogram model demonstrated good stability and clinical net benefit after internal bootstrap validation, it still lacks external validation with a large sample size from multiple centers, which may preclude its broad application. Therefore, our main focus in the future will be to increase our sample size by collecting data from multiple centers to improve and externally validate the stability and broad applicability of our nomogram risk prediction model. Ultimately, we anticipate that this model will provide clinicians with a rapidly accessible and reliable clinical scoring tool for the individualized prevention, treatment, and prognostic improvement of no-reflow after primary PCI in high-risk patients with STEMI.

## Conclusion

In this study, a nomogram risk prediction model for the occurrence of no-reflow after primary PCI in patients with STEMI was constructed. The model is based on rapidly accessible clinical data, including Killip class on admission, fibrinogen and D-dimer levels, and SII. It demonstrated good discrimination, calibration, and clinical validity, as well as superior predictive performance for no-reflow after primary PCI compared with traditional predictors such as Killip class ≥2 on admission, plasma D-dimer and fibrinogen levels, and SII. Our findings further demonstrated that the total score of the nomogram risk prediction model demonstrated good predictive performance for in-hospital and 1-year post-discharge MACE after primary PCI among patients with STEMI.

## Data availability statement

The data analyzed in this study is subject to the following licenses/restrictions: None. Requests to access these datasets should be directed to 13685533763@163.com.

## Ethics statement

The studies involving human participants were reviewed and approved by The 904th Hospital of Joint Logistic Support Force of PLA. Written informed consent was obtained from the patients and their family members before the primary PCI procedure.

## Author contributions

GZ, YL, and TY designed the study. KC and GW performed the statistical analysis. YL drafted the manuscript. All authors gave comments and suggestions, and approved publication.

## Funding

This work was supported by the Major topics of the health commission of Jiangsu Province (ZD2021020), Key topics in medical and health of Wuxi Bureau of science and technology, Jiangsu Province (Y2021011), and Supported by Medical Key Discipline Program of Wuxi Health Commission (CXTD2021008).

## Conflict of interest

The authors declare that the research was conducted in the absence of any commercial or financial relationships that could be construed as a potential conflict of interest.

## Publisher's note

All claims expressed in this article are solely those of the authors and do not necessarily represent those of their affiliated organizations, or those of the publisher, the editors and the reviewers. Any product that may be evaluated in this article, or claim that may be made by its manufacturer, is not guaranteed or endorsed by the publisher.
