# Corrigendum: A nomogram risk prediction model for no-reflow after primary percutaneous coronary intervention based on rapidly accessible patient data among patients with ST-segment elevation myocardial infarction and its relationship with prognosis

**DOI:** 10.3389/fcvm.2023.1229889

**Published:** 2023-06-19

**Authors:** Yehong Liu, Ting Ye, Ke Chen, Gangyong Wu, Yang Xia, Xiao Wang, Gangjun Zong

**Affiliations:** ^1^ Department of Cardiology, The 904th Hospital of Joint Logistic Support Force of PLA, Wuxi Clinical College of Anhui Medical University, Wuxi, China; ^2^ The Fifth Clinical College of Anhui Medical University, China

**Keywords:** no-reflow, percutaneous coronary intervention, ST-segment elevation myocardial infarction, nomogram risk prediction model, prognosis, major adverse cardiovascular events

A Corrigendum on A nomogram risk prediction model for no-reflow after primary percutaneous coronary intervention based on rapidly accessible patient data among patients with ST-segment elevation myocardial infarction and its relationship with prognosis By Liu Y, Ye T, Chen K, Wu G, Xia Y, Wang X, Zong G. Front Cardiovasc Med. (2022) 9:966299. doi: 10.3389/fcvm.2022.966299

**Incorrect Affiliation**

In the published article, there was an error in affiliations 1 and 2.

[1] Instead of “[Department of Cardiology, The 904th Hospital of Joint Logistic Support Force of PLA, Wuxi, China]”, it should be “[Department of Cardiology, The 904th Hospital of Joint Logistic Support Force of PLA, Wuxi Clinical College of Anhui Medical University, Wuxi, China]”.

[2] Instead of “[Wuxi Clinical College of Anhui Medical University, Wuxi, China]”, it should be “[The Fifth Clinical College of Anhui Medical University, China]”.

The authors apologize for this error and state that this does not change the scientific conclusions of the article in any way. The original article has been updated.

